# State of the literature discussing smoke-free policies globally: A narrative review

**DOI:** 10.18332/tid/174781

**Published:** 2024-01-05

**Authors:** Jacqueline A. Teed, Meagan O. Robichaud, Michelle Duren, Hebe N. Gouda, Ryan David Kennedy

**Affiliations:** 1 Department of International Health, Johns Hopkins Bloomberg School of Public Health, Johns Hopkings University, Baltimore, United States; 2 Department of Health Behavior and Society, Johns Hopkins Bloomberg School of Public Health, Johns Hopkings University, Baltimore, United States; 3 Department of Health Policy and Management, Johns Hopkins Bloomberg School of Public Health, Johns Hopkings University, Baltimore, United States; 4 Tobacco Free Initiative, World Health Organization, Geneva, Switzerland; 5 Institute for Global Tobacco Control, Bloomberg School of Public Health, Johns Hopkins University, Baltimore, United States

**Keywords:** tobacco, review, smoke-free policy, LMICs, HICs

## Abstract

Despite the success of the Framework Convention on Tobacco Control (FCTC), most jurisdictions in the world do not have policies that create 100% smoke-free environments in indoor workplaces, indoor public places, public transport, or other public places. We conducted a narrative review of articles that discuss smoke-free policies and describe the state of the current literature. A search of peer-reviewed and gray literature, published between 1 January 2004 and 30 April 2022, was conducted using PubMed and EMBASE databases. We classified articles based on the location of the policy discussed (WHO region, World Bank income classification) and the environment that was being made smoke-free. Insights related to policy development and implementation, as well as compliance and enforcement, were also identified. The search identified 4469 unique citations; 134 articles met the criteria for inclusion and underwent data extraction by two independent coders. The sample included articles published in or about jurisdictions in each WHO region, in high- and low- and mediumincome countries, and articles that discussed policies regulating smoke-free indoor workplaces, indoor public places, public transport, outdoor/quasi-outdoor environments, and other (unspecified) public places. Some important insights from the literature related to smoke-free policy implementation included tobacco industry interference, the important role of civil society, and the need for effective communication, education, and leadership. Enforcement officials’ awareness and training, stakeholders’ attitudes and beliefs, and understanding social norms were identified as relevant determinants of effective smoke-free policies. There continue to be challenges for implementing smoke-free policies in jurisdictions throughout the globe, in high- and low- and middle-income countries. The literature includes insights to support 100% smoke-free policies in each environment that must be made smoke-free as per the FCTC.

## INTRODUCTION

Tobacco use is responsible for more than 8 million deaths per year, with 1.3 million of these deaths attributable to secondhand smoke (SHS) exposure^1^. SHS exposure causes many negative health outcomes, including cardiovascular disease and cancer^2^. Comprehensive smoke-free laws are an important public health strategy because they reduce tobacco smoke exposure and are associated with a decrease in youth smoking initiation^3,4^.

The Framework Convention on Tobacco Control (FCTC) is one of the most successful global treaties with 182 Parties ratifying the convention as of July 2023^5,6^. Article 8 of the FCTC obligates Parties to implement comprehensive smoke-free laws in all ‘indoor workplaces, public transport, indoor public places and, as appropriate, other public places’, which may include outdoor or quasi-outdoor environments^5,7^. Guidelines for the implementation of Article 8 were adopted in 2007 and outline steps for Parties to follow to ensure the development of effective smoke-free policies^7^.

A 2019 review conducted by Byron et al.^8^ identified ongoing challenges in the effective implementation, compliance, and enforcement of smoke-free legislation in low- and middle- income countries (LMICs) following FCTC ratification, citing obstacles such as limited accountability and weak implementation strategies. The review examined the literature published until January 2017, and proposed a research agenda intended to support governments to implement effective smoke-free policies in LMICs, including identifying the critical lessons learned for effective implementation, evaluating different enforcement approaches, rejuvenating stalled smoke-free policies, and increasing political will to enforce policies^8^.

The 2023 WHO Report on the Global Tobacco Epidemic reports on the state of smoke-free policies for each of the 202 member states of the WHO and identified comprehensive smoke-free policies in 74 countries, which are protecting an estimated 2.1 billion people^9^. These Parties have policies that meet the obligations of Article 8 and have created smoke-free environments through national policies or have at least 90% of their population protected from SHS through sub-national smoke-free policies^8^. This represents tremendous progress; however, 128 jurisdictions, including 42 highincome countries, 66 middle-income countries, and 20 low-income countries, lack comprehensive smoke-free policies, leaving most of the world’s population unprotected or only partially protected from the dangers of SHS^8^. Jurisdictions from all income classifications are clearly facing challenges implementing comprehensive smoke-free laws.

The present review seeks to report on the state of the literature discussing country-level smoke-free policies from jurisdictions from all income classifications. In this review, we characterize the literature identified based on where smoke-free policies are discussed (which WHO region), as well as the number of articles that discuss smoke-free policies in LMICs and HICs according to the World Bank’s income classification^10,11^. This review reports the number of studies that focused on smoke-free policies in specific environments detailed in the implementation guidelines for Article 8, including indoor workplaces, indoor public places, transport, outdoor/quasi-outdoor environments, and other public places. This study further identifies lessons from smoke-free policy development and implementation, as well as lessons related to policy compliance and strategies for enforcement.

A literature search of academic and gray literature published between 1 January 2004 through 30 April 2022, was conducted through PubMed and EMBASE databases. Search terms included combinations and variations of: [implementation OR enforcement OR compliance] AND [smoke-free OR ban OR restriction] AND [tobacco OR smoking]. The team used Covidence to manage the identified citations. Covidence is a web-based collaboration software platform that streamlines the production of systematic and other literature reviews^12^. The full search strategy is provided in the Supplementary file.

Articles were included in the study if they contained content about smoke-free policies or circumstances that impact smoke-free policies in at least one of the following settings listed in Article 8 of the FCTC: indoor workplaces, indoor public places, public transport, outdoor or quasi-outdoor environments, or other (unspecified) public places. Articles that focused on other smoke-free environments (e.g. prisons or multi-unit housing), that assessed voluntary smoke-free policies, that were primarily about other tobacco control policies or interventions (e.g. smoking cessation), or that had been previously identified and reviewed by Byron et al.^8^, were excluded. Commentaries, clinical studies, and non-English articles were also excluded.

**Figure 1:**
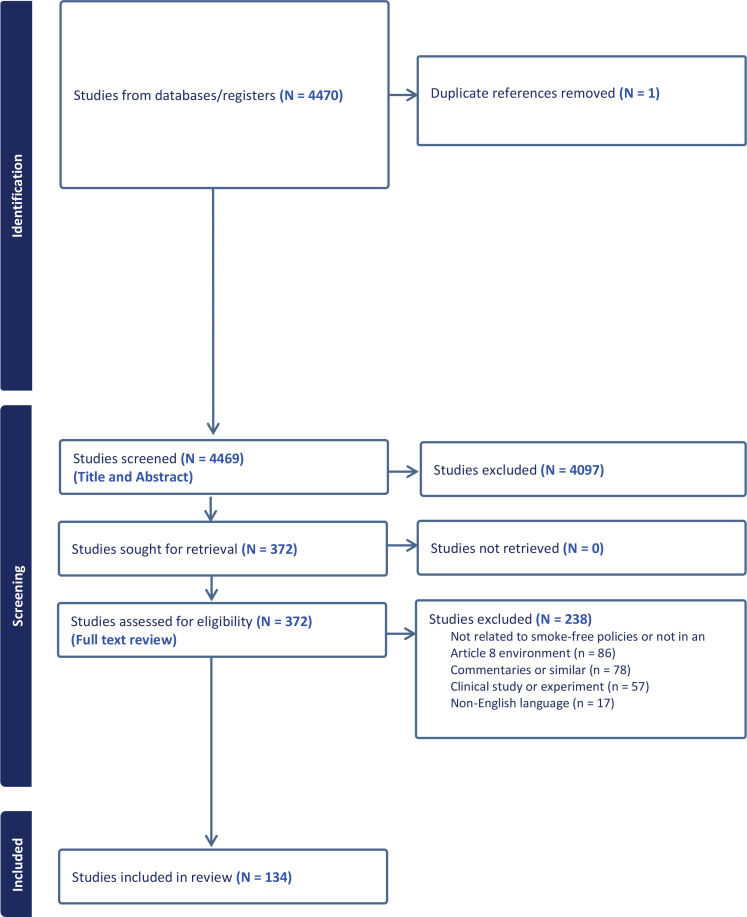
PRISMA flowchart showing the number of citations identified at each stage in the search and screening process

The search produced 4470 articles. One duplicate article was removed. The titles and abstracts of 4469 articles were screened by two independent coders. Of these, 372 articles met the inclusion criteria and underwent full-text review by the two coders. After the full-text review, 238 articles were excluded because they did not present findings relevant to smoke-free policies or Article 8 environments (86 articles), were commentaries or similar (78 articles), reported results of a clinical study or experiment (57 articles), or were not published in English (17 articles). The search ultimately identified 134 articles that met the criteria for inclusion (Figure 1).

These 134 articles were reviewed, and the country or sub-national jurisdiction where the smoke-free policies were enacted was noted and classified by its respective WHO Regional Office: Regional Office for Africa (AFRO), Regional Office for the Americas (AMRO), Regional Office for the Eastern Mediterranean (EMRO), Regional Office for Europe (EURO), Regional Office for South-East Asia (SEARO), and Regional Office for the Western Pacific (WPRO). Countries/jurisdictions were also classified according to the World Bank’s income classifications^11^. Although the World Bank classifies countries as low-income, middle-income, upper middle-income, and high-income, the study team classified the articles by three categories: LMIC-specific, HIC-specific, or both LMIC and HIC^11^. The environment(s) that the policy focused on making smoke-free was noted (indoor public places, indoor workplaces, public transport, outdoor and quasi-outdoor environments, and/or unspecified). In some cases, articles discussed policies in multiple jurisdictions and/or discussed multiple relevant environments.

The study team also classified articles based on their content. A priori codes related to the smoke-free policy themes included policy development, implementation, enforcement, and compliance. The study team defined each a priori code as follows:

- Development: The process of formulating any mandatory law(s) and/or regulation(s) to establish smoke-free environment(s) in indoor public places, indoor workplaces, public transport, outdoor and quasi-outdoor environments, and/or other (unspecified) public places. We considered policy development for both partial and complete smoking bans.
- Implementation: The process of introducing and integrating any mandatory smoke-free policy(ies) within indoor public places, indoor workplaces, public transport, outdoor and quasi-outdoor environments, and/or other (unspecified) public places. We considered implementation to include any initial and/or ongoing practical measure(s), strategy(ies), and/or administrative action(s) that worked toward putting smoke-free policies into place and establishing partially or completely smoke-free environment(s).
- Enforcement: The application of any formal or informal legal and/or regulatory measure(s), monitoring mechanism(s), and/or strategy(ies), upheld by any individual(s) or group(s), to ensure adherence to the requirements of the smoke-free policy(ies) and smoke-free environment(s) in indoor public places, indoor workplaces, public transport, outdoor and quasi-outdoor environments, and/or other (unspecified) public places.
- Compliance: The degree to which individuals, communities, businesses, workplaces, and/or any other public place(s) or public institution(s) adhered to the smoke-free policy’s requirements in indoor public places, indoor workplaces, public transport, outdoor and quasi-outdoor environments, and/or other (unspecified) public places.

After initial extraction, the articles were reviewed a second time, and the research team reached consensus on the themes and extracted relevant examples and quotes from each article that corresponded to these themes. The team then synthesized insights across the sample of articles to present examples.

The Supplementary file provides a table with a description of the included studies. The table presents the first author and publication year of the study, the country(ies)/jurisdiction(s) where the study was conducted, the World Bank income classification(s) of the country(ies)/jurisdiction(s), the WHO region(s) to which the country(ies)/jurisdiction(s) belong, the environment(s) studied, and the main theme(s) explored in the study. The citation for each study is also provided.

## GLOBAL SMOKE-FREE POLICIES

### WHO regions and World Bank income classifications

The number of articles by WHO region and World Bank income classification is presented in Table 1. The study identified 40 articles that discussed smoke-free policies in EURO, 30 articles in AMRO, 21 articles in SEARO, 16 articles in WPRO, 8 articles in EMRO, 7 articles in AFRO. Τwelve articles discussed smoke-free policies within multiple WHO regions. The sample of articles included 79 articles discussing smoke-free policies in HICs, 41 articles discussing smoke-free policies in LMICs, and 14 articles discussing both LMICs and HICs.

**Table 1:** Number of articles by World Health Organization regional office and World Bank income classification (N=134)

*Regions and income classifications*	*Articles n (%)*
**WHO regional office**	
AFRO	7 (5.2)
AMRO	30 (22.4)
EMRO	8 (6.0)
EURO	40 (29.9)
SEARO	21 (15.7)
WPRO	16 (11.9)
Multiple regions	12 (9.0)
**World Bank income classification**	
HIC-specific	79 (59.0)
LMIC-specific	41 (30.6)
Both LMIC and HIC	14 (10.4)

AFRO: Regional Office for Africa. AMRO: Regional Office for the Americas. EMRO: Regional Office for the Eastern Mediterranean. EURO: Regional Office for Europe. SEARO: Regional Office for South-East Asia. WPRO: Regional Office for the Western Pacific. HIC: high-income countries. LMIC: low- or middle-income countries.

### Smoke-free environments

The number of articles discussing each environment is outlined in Table 2. This study identified articles discussing smoke-free policies in each of the environments required to be made smoke-free as detailed in the Guidelines for Article 8 of the FCTC, including indoor public spaces (102 articles), indoor workplaces (98 articles), outdoor and quasi-outdoor environments (46 articles), public transport (29 articles), and unspecified public places including outdoor or quasi-outdoor environments (13 articles).

**Table 2:** Number of articles by environment and main theme (N=134)

*Environment and Themes*	Articles n (%)*
**Environment**	
Indoor public places	102 (76.1)
Indoor workplaces	98 (73.1)
Public transport	29 (21.6)
Outdoor and quasi-outdoor environments	46 (34.3)
Unspecified	13 (9.7)
**Main theme**	
Development	67 (50.0)
Implementation	119 (88.8)
Compliance	78 (58.2)
Enforcement	75 (56.0)

* Multiple environments and main themes could be applied to the same article.

### Themes and insights discussed in articles

Table 2 presents the number of articles discussing smoke-free policy development, implementation, enforcement, and compliance. The following paragraphs summarize key insights from our review of the literature. The research team observed a high level of overlap between issues affecting policy development and policy implementation, as well as those affecting compliance and enforcement. Therefore, the results are presented in two main sections (policy development and implementation; policy compliance and enforcement). Specific barriers and opportunities are discussed throughout both main sections.

*Policy development and implementation*

In all, 67 articles discussed the development of smoke-free policies, and 119 articles discussed policy implementation. Stakeholder involvement was the most commonly identified factor influencing smoke-free policy development and implementation. The literature further coalesced on two sides regarding stakeholder involvement in smoke-free policy – the influence of tobacco industry interference and the importance of civil society engagement. In addition to stakeholder involvement, several studies discussed the importance of communication and educational efforts and effective leadership. Finally, access to resources was an additional factor identified in the literature as an important determinant of smoke-free policy development and implementation.

*Tobacco industry interference*

Many studies explicitly mentioned tobacco industry interference as a significant barrier to enactment and implementation of smoke-free policies within both HICs and LMICs^13-33^. Studies have linked tobacco industry interference to delayed legislation, weak draft bills, improper policy adoption, and limited and ineffective policy implementation^23,24,34,35^. Government officials vary in their attitudes toward interactions with the tobacco industry, and there is sometimes limited awareness among government officials with regard to Article 5.3 of the FCTC^22^. This lack of knowledge, combined with fear of the tobacco industry, poses a significant barrier to implementing comprehensive smoke-free policies. The tobacco industry uses a variety of tactics to prevent or delay the enactment of comprehensive smoke-free policies or to undermine existing smoke-free policies. In addition to directly lobbying government officials to block or weaken smoke-free policies^15,23,24,26,29,30,32^ and using front groups to advance their interests^15,17,21,23,24,26,32,36,37^, the tobacco industry has challenged smoke-free policies through litigation^13,23,24,30,32,37^. For example, in Kenya, the tobacco industry challenged the Tobacco Control Act of 2007 in court, resulting in the suspension of smoke-free policies^30^. The tobacco industry has also promoted weak smoke-free policies to prevent enactment of comprehensive smoke-free policies^18,23,24,30^. For example, across multiple countries, tobacco companies (including British Americand Tobacco and Philip Morris International) promoted the ‘Courtesy of Choice program’ to encourage hospitality venues to self-regulate and provide smoking and non-smoking areas^30,32^. Additional tobacco industry interference tactics include promoting positive perceptions of the tobacco industry and manipulating and misrepresenting evidence to decision-makers^38^.

In addition to economic arguments, the tobacco industry has attempted to appeal to social or cultural norms and values, such as individual rights or freedom. One study of street smoking bans in Japan found that many municipalities permit smoking in streets as long as an ashtray is provided or the smoker carries a portable ashtray to dispose cigarette butts^18^. The authors suggested these partial outdoor smoking bans reflect tobacco industry marketing strategies that emphasize cleanliness and ‘proper etiquette’ (e.g. not littering), with the underlying premise that smoking is acceptable within these constraints – and further finding that tobacco interest groups used these outdoor restrictions to oppose indoor smoking restrictions^18^.

*Civil society engagement*

The literature frequently identified civil society engagement as a specific form of stakeholder involvement that was an effective strategy for successful smoke-free policy development and implementation^9,18-20,23,24,37,39-51^. Involving public health officials^30,43,44^, researchers^51^, community groups^37,43,45,51^, journalists^23,24,43^, and those most affected by the policy^40,42,46^ was found to enhance the policymaking process. A weak or fragmented civil society was identified as a barrier to smoke-free policy implementation in Latin America^32^. Stakeholder involvement was critical at every stage of the policymaking process, including policy formulation^23,24,40^, adoption^52,53^, implementation^46,47^, and evaluation^51^. Media pressure and engagement were highlighted as ways to increase civic support, awareness, education, youth advocacy, and stakeholder engagement^18,23,24,26,27,34,36,37,54-63^. Civil society organizations can also help countries monitor and counter tobacco industry interference, and build capacity for implementing and evaluating smoke-free policies^23,24^. Additionally, civil society can engage in litigation and lobbying to advance tobacco control efforts. For example, after the tobacco industry filed cases in the Supreme Court against a comprehensive tobacco control law in Nepal, the civil society groups, Action Nepal, and Health and Environment Awareness Forum Nepal, met with health professionals, lawyers, and media groups to increase awareness of this issue and also lobbied Nepal’s Attorney General to hasten the hearing^23,24^. Additionally, civil society groups in Nepal filed a case against the government in the Supreme Court to pressure for immediate implementation of comprehensive tobacco control policies^23,24^.

*Government engagement*

Engagement from all levels of government, including national, state/provincial, and local governments, can contribute to successful policy development and implementation, particularly in countering tobacco industry interference^22-24^. One study emphasized the importance of ‘bottom-up’ approaches, in which local or district-level policies and actions can encourage action from state/provincial or national governments by serving as role models^22^. Given that the tobacco industry uses similar interference tactics globally, governments and tobacco control advocates can anticipate opposition strategies from the tobacco industry and plan their response in advance^32^. Several countries have developed national plans, strategies, and innovative approaches to counter tobacco industry interference, including ratifying the FCTC and implementing provisions specified in Article 5.3^25-27,32,34,37,51,64,65^. The literature also emphasized the importance of increasing awareness and compliance with FCTC Article 5.3 in countering tobacco industry interference^22^. For example, following efforts to sensitize health officials to obligations under Article 5.3, health officials in Bangladesh discontinued engagement with the tobacco industry^22^. One study suggested that raising awareness of Article 5.3 across different sectors, not just the health sector (i.e. horizonal coordination), as well as cooperation between national, state/provincial, and local governments (i.e. vertical cooperation) was important for successfully countering tobacco industry interference^24^.

*Effective leadership*

Effective leadership was another key factor identified in the literature, including leadership from government and civil society^9,23,24,36,37,40,46,47,49,54,66-69^. Leaders provide a central authority and convene disparate stakeholder groups^20,69^. Effective leaders also serve as champions and positive advocates for smoke-free policies, thereby increasing support and buy-in^69^. For example, Kansas City (Missouri, United States) had clear, identifiable policy champions, who had extensive networks and worked to actively connect different stakeholder groups, ‘including the media, coalitions, public health agencies, policymakers, and other partners’^69^. These policy champions ‘built a case for non-smokers’ rights’ and were instrumental in moving a smoke-free policy forward^69^. Stakeholders were well-connected (not siloed), and all stakeholders actively engaged and communicated throughout the policy process^69^. For smoke-free policies directed at hospitals, schools, or similar institutions, involvement of senior management was consistently identified as a crucial component to successful implementation of smoke-free policies^40,66,68,70-72^.

*Communication, education, and training*

Limited awareness of the dangers of SHS and of existing policies, even among policymakers, can be a barrier to policy implementation^27,28,34^. Clear, effective communication and education about the dangers of SHS and about smoke-free policies was discussed in many studies as key to successful policy implementation^23,24,31,32,34,36,42-44,46,47,50,53,55,60,69,73-79^. For example, public colleges and universities in New Zealand utilized several communication strategies to improve smoke-free policy implementation, including signage, providing information about the smoke-free policies during orientation and in student handbooks, local media publicity, providing information through websites and social media, and sharing positive comments from students and staff about the policy^46^. The literature also identified communication between stakeholder groups as an important contributor to the development and implementation of smoke-free policies^20,39,72,80^.

In addition to raising awareness about the need for smoke-free policies, clear communication and education can potentially increase support and buy-in for smoke-free policies from governments, businesses, institutions, and the public. This support, or lack thereof, can impact the success of smoke-free policy implementation^39,43,44,46,47,81-84^. One factor that can negatively impact support from policymakers is perceived public opposition to smoke-free policies^39,44,46,81-84^. Given that policymakers often underestimate the level of public support for smoke-free policies, fear of public opposition can be a barrier to successful implementation^39,44,46,81-84^. For example, studies suggest there is strong public support for implementing at least some smoking restrictions in India, with 98% of respondents from India in a global study supporting public smoking bans^83^. However, in a sample of representatives from local self-government bodies in two districts in the state of Kerala, 25% reported fear of public opposition as a major barrier to implementing FCTC provisions, including smoke-free policies^83^. These findings suggest that educational efforts are needed to address policymakers’ concerns about public opposition^83^.

Furthermore, several studies discuss the lack of training for those tasked with implementation as a barrier to successful smoke-free policy implementation^40,66,68,73,85,86^. In particular, staff in healthcare settings (including psychiatric units) often lack training to deliver smoking cessation services to patients who smoke, which is an important component of successful smoke-free policy implementation in these settings^40,66,68,73,85,86^. For example, a study of 192 psychiatric service centers in Catalonia, Spain, found that only 27.5% provided information briefings on how to implement smoke-free policies, and only 37.9% had ‘smoking intervention training’ available to staff^85^. Thus, training for stakeholders tasked with implementation of smoke-free policies can potentially increase the success of smoke-free policies.

*Access to resources*

Access to adequate resources, including financial, technical, and legal resources, was a key policy development and implementation factor identified in the literature, with the lack of resources identified as a barrier to successful policy implementation^25,32-34,51,52,76^. The need for financial resources to aid policy implementation was most frequently identified in LMICs^37^. Limited access to information, including smoking prevalence data, was a related constraint identified^25^. One study found that the lack of reliable and complete data on smoking prevalence information in Sub-Saharan African countries was a limiting factor for policy development; such data could be an important agenda-setting driver, providing evidence of smoking as a problem needing to be addressed^25^. Support from non-governmental organizations (NGOs) can mitigate resource constraints through grants, training, and technical support. For example, in Latin America, the Pan American Health Organization (PAHO) launched the Smoke-Free Americas Initiative in 2001, organized training workshops for tobacco control advocates and decision-makers, and provided seed grants to support smoke-free campaigns^32^. Grants from universities, NGOs and government agencies (e.g. Johns Hopkins University, Canadian International Development Research Center) also supported implementation of the FCTC and trained researchers throughout Latin America^32^. In December 2020, South America became the first subregion in the Americas to have 100% smoke-free environments in line with Article 8 of the FCTC^87^. Additionally, earmarked tobacco taxes (e.g. from cigarette taxes) could be used to fund tobacco control efforts such as smoke-free policies^64^. One study suggested that ‘user fees’ on the tobacco industry could also provide funding to support tobacco control efforts^64^.

### Policy compliance and enforcement

Following successful implementation of a smoke-free policy, challenges may remain for reaching full compliance^21,67,82,88^. This study identified 78 studies that discussed compliance with smoke-free policies and 75 studies that discussed enforcement. Key factors identified in the literature as inhibiting smoke-free policy compliance include: a lack of awareness and training among stakeholders, limited government involvement in policy implementation and enforcement, lack of resources, negative attitudes and perceptions of smoke-free policies, and social and cultural norms^21,44,46,59,81,89-92^. To combat these challenges, studies recommend educational efforts, stakeholder involvement, and taking action to address social norms^27,74,78,81,93-99^.

*Awareness of smoke-free policies and dangers of SHS*

Limited awareness of smoke-free policies, including where smoking is prohibited, as well as the dangers of tobacco use and SHS, is a key barrier to policy compliance and enforcement^27,31,47,50,100,101^. Several studies found that the required ‘no-smoking’ signage was often absent in places where smoking is prohibited, potentially contributing to a lack of awareness of smoke-free policies in these settings^27,74,102-104^. For example, an observational study conducted in Pakistan reported the presence of no-smoking signage at the main entrance and inside for only 6% and 10% of places, respectively^103^. Additionally, one study in Thailand suggested that there was limited awareness of the dangers of SHS among the public^27^. Several studies recommend clear communication and educational efforts to improve knowledge about the dangers of SHS and to increase awareness of the smoke-free policies and of the penalties for non-compliance^27,48,55,63,68,72-74,81,82,94,97,102,104-107^. These efforts can include educational campaigns through various media channels, peer education, and no-smoking signage specifying penalties for non-compliance^27,48,55,63,68,72-74,81,82,94,97,102,104-107^. For example, representatives from civil society organizations in Uganda suggested communicating specific policy details (e.g. distance of buffer around smoke-free places, specific penalties, signage requirements, inclusion of shisha use in the smoke-free policy), roles and responsibilities of venue staff, and the health risks of smoking^82^. Stakeholders also emphasized the need to translate educational materials into local languages and to use multiple communications channels (e.g. radio, in-person meetings) to reach people with lower levels of literacy^82^. Comprehensive education programs surrounding the dangers of tobacco use is an approach found to improve compliance among schools in rural India^72^. However, one study of campus smoke-free policies in Sudan found that most university students who smoked cigarettes or hookah, or who dipped tombac, were aware of their school’s smoke-free policies^92^, and Wynne et al.^74^ described a study from Greece suggesting that the presence of ‘no-smoking’ signage did not impact levels of SHS in some locations. These findings suggest that awareness is an important step but is insufficient on its own to improve compliance with smoke-free policies.

*Stakeholder training*

In addition to limited knowledge about smoke-free policies and the dangers of SHS, several studies cited a lack of training for stakeholders responsible for policy enforcement (e.g. law enforcement, hospital staff) as a barrier to enforcing smoke-free policies^21,27,44,108^. For example, one study of middle managers in a private company in Denmark found that, while middle managers supported smoke-free policies, they did not feel they were responsible for ‘regulating other peoples’ actions’ and did not feel adequately prepared to address violations and discuss smoking cessation with their employees^108^. Thus, training for enforcement stakeholders that discusses the extent of their roles and responsibilities, protocol for responding to violations, and (in healthcare settings) providing smoking cessation services, can potentially increase compliance^27,44,66,68,73,74,95,108,109^.

*Attitudes, beliefs, and perceptions about smoke-free policies*

Stakeholders’ attitudes, beliefs and perceptions about smoke-free policies and their outcomes can significantly impact the success of smoke-free policy compliance and enforcement. Among businesses and institutions, perceptions of public support or opposition to smoke-free policies may contribute to willingness or hesitancy to comply with smoke-free policies^84^. These attitudes, beliefs, and perceptions can lead to a lack of organizational, administrative, and managerial buy-in and support, which can be a barrier to compliance and enforcement of smoke-free policies^16,50,66,73,84,110^. One study found that those charged with implementing smoke-free policies for bars and gaming venues in Australia significantly underestimated community support for smoke-free policies, suggesting that demonstrated community support could aid compliance and enforcement^84^. Additionally, the misperception that smoke-free laws will negatively impact businesses, particularly the hospitality industry and bars, can be a barrier to enforcing smoke-free policies, suggesting there is a need to address these misperceptions through educational efforts^47,82,84^.

Several studies also found that some institutions, businesses, or stakeholders do not enforce smoke-free policies because it is a low priority amid competing needs and interests^39,44,52,53,73,110,111^. For example, police at public universities in California stated that enforcing tobacco-free policies on campus was not a priority because they ‘have real crimes [such as armed robbery] to investigate’^44^. Resource constraints, including limited money, time, and personnel, may further exacerbate this issue and contribute to low prioritization of enforcing smoke-free policies^29,33,37,39,44,50,72,89,104,110-113^. For example, a study conducted in schools across multiple European cities found that teachers and principals, who already face time constraints and have competing priorities, may be unable or unwilling to accept additional responsibilities related to enforcing smoke-free school policies^39^.

Additionally, some enforcement stakeholders believed that enforcing smoke-free policies would interfere with more important goals and values^70,111^. For example, school principals in Sweden expressed concern that punishing students (e.g. especially through suspension or expulsion) for violating their schools’ smoke-free policies would interfere with the more important goal of students staying in school and learning^111^. In mental healthcare settings, some staff and management were opposed to smoke-free policies because they were concerned that such polices would take away more freedoms from patients whose freedom of choice is already being restricted^70^. Staff and management were also concerned that focusing on smoking cessation would detract from addressing patients’ primary mental health concerns^70^. In settings where current enforcement mechanisms conflict with these other goals, stakeholders can consider adopting different strategies to improve compliance, such as initiating dialogue and counselling instead of more punitive measures^111^. Furthermore, communication and education efforts should emphasize that smoke-free policies promote the well-being of those impacted by these policies. For example, to address stakeholders’ beliefs about patients’ right to smoke, communication efforts can emphasize patients’ right to a safe and health-promoting environment (i.e. as patients in other healthcare settings receive) and discuss how smoke-free policies work to reduce existing health disparities that impact people with severe mental illness by reducing premature mortality in this population^70^.

*Government involvement*

Achieving high levels of policy compliance is challenging without clear, specific guidance from national, state/provincial, and local governments^21,44,74,81,82,91,112^. For example, local governments in California, tasked with enforcing the state’s smoke-free law, were only provided with a penalty schedule for violations (i.e. a $100 maximum fine for a first violation) and were not given specific frameworks or mechanisms for enforcing smoke-free policies^112^. Local governments were given discretion to determine who was tasked with enforcing the smoke-free policies, stating only that the policy ‘was to be enforced by local law enforcement agencies, including, but not limited to, local health departments’^112^. Additionally, local administrative structures were described as ‘administrative maze[s]’, leading to a lack of clarity regarding roles and responsibilities and inconsistent enforcement^112^. Several articles highlighted the need for legislation to incorporate a formal enforcement mechanism with clear guidelines on enforcement recommendations and expectations^44,74,82^. Additionally, some studies highlighted a lack of funding from state or national governments for carrying out enforcement tasks as a barrier to enforcing smoke-free policies^27,112^. Limited legal infrastructure in certain countries poses an additional enforcement challenge^63^.

*Norms*

Another barrier to policy compliance and policy enforcement found in the literature is the issue of ingrained social norms and the need to develop tailored, culturally appropriate tobacco policy to specific jurisdictions and settings^16,27,29,48,66,68,73,79,89,90,101,114,115^. These norms can include a persistent ‘smoking culture’ in certain settings, such as bars or psychiatric units, as well as other social norms, such as cigarette sharing or gifting (a common social practice in China) and avoiding interpersonal conflict, which can be barriers to compliance and enforcement^66-68,73,79,101,114,115^. Such norms, combined with a lack of managerial support and a norm supporting the social acceptability of smoking, make it difficult for smoke-free policies to be accepted and enforced in many settings^22,67,83,116^. For example, some bar and club patrons and owners may suggest that smoking is an integral part of the experience in these venues, and this belief poses barriers to implementing and enforcing smoke-free policies in these settings^114,115^. The literature discusses several potential strategies for addressing these social norms^27,44,48,81,102^. These strategies include communication and education campaigns that aim to decrease the acceptability of tobacco use and promote a tobacco-free lifestyle^44,48,81^. Communication efforts can include messaging that extends accepted non-smoking scenarios, such as the idea that ‘exposing adults to toxic smoke is no more appropriate than exposing children to it’^48^. Other studies suggest removing smoking-cues, such as ashtrays or cigarette butts, which may indirectly communicate the social acceptability of smoking in venues^48,75,102^. In settings where stakeholders are reluctant to engage in confrontation to address violations of smoke-free policies, one study suggested reframing enforcement efforts as ‘teachable moments’, rather than viewing these efforts as confrontational^27^.

*Community engagement*

In addition to formal enforcement mechanisms (e.g. police enforcing smoke-free policies), some studies discussed engaging community members to improve compliance and to aid enforcement efforts^27,43,44,55,75^. For example, the Beijing Tobacco Control Association launched ‘The Complaint Map’, in which users could report violations of smoke-free policies via WeChat and recruited tobacco control volunteers to help respond to violations of smoke-free policies^75^. Location services were used to generate a map of violations to identify where and when violations most frequently occur, allowing for more targeted and efficient enforcement^75^. Participatory processes for developing enforcement mechanisms, which involve stakeholders most impacted by smoke-free policies (e.g. business owners and patrons), can potentially improve support for smoke-free policies and improve compliance^79^. For example, key informants from traditional villages in Denpasar, Bali, suggested developing smoke-free policies through *Pararem* (‘local wisdom or local policy … generated through community meeting and agreement’ that include ‘social sanctions’) to improve buy-in and compliance among indigenous Balinese people who are members of the *desa adat* (traditional village system)^79^.

## DISCUSSION

This review builds on the previous work of Byron et al.^8^ to provide an overview of the state of literature discussing smoke-free policies. The current review found published work discussing smoke-free policies from each World Bank income strata and WHO region of the world, and it highlighted some key barriers and facilitators to smoke-free policy development, implementation, compliance, and enforcement. Additionally, this study identified specific factors as critical to determining the effective development and implementation of smoke-free policies, including tobacco industry interference; civil society engagement; government engagement; leadership; communication, education, and training; and access to resources. Across studies, smoke-free policy compliance and enforcement were most impacted by stakeholders’ levels of awareness of smoke-free policies and the dangers of SHS; stakeholder training; government involvement; stakeholders’ attitudes, beliefs, and perceptions; social norms within the community; and level of stakeholder engagement.

Tobacco industry interference continues to be one of the most substantial, overarching barriers to each aspect of successful smoke-free legislation. Several articles included in this review mentioned a variety of industry tactics, such as country-specific litigation efforts, the promotion of weak (not comprehensive) smoke-free policies, and political manipulation, that aim to delay or prevent comprehensive smoke-free policies or undermine existing ones^23,24,34,35^. Similar to previous studies, this review found governments in LMICs to be particularly vulnerable to tobacco industry interference, although some governments in HICs also faced challenges^13-33,38^. Because the tobacco industry employs similar strategies globally, it may be beneficial for governments to work with civil society organizations to anticipate industry opposition and preemptively plan their response using previously successful mitigation strategies. While these mitigation strategies need to be adapted to specific cultural contexts and social milieus, the strategies presented in this review may provide a basic foundation for countries seeking to counter tobacco industry interference.

For example, in addressing tobacco industry interference, ‘bottom-up’ policy approaches tended to be quite successful in certain settings^22^. Based on the literature, it is possible that locally implemented policies may be more effective in certain settings due to the feasibility of implementation and the lack of attention from national tobacco lobbies. Because the tobacco industry has the ability to obstruct the successful implementation of national laws in many countries, certain subnational jurisdictions may be forced to implement smoke-free legislation as they can. Although this finding underscores the need to eliminate global tobacco industry interference and develop national plans to counter such interference, it also highlights the ability of local governments to successfully enact their own tobacco control efforts. To support the development of more comprehensive and sustainable tobacco control strategies, it is essential to acknowledge the power of local smoke-free policies while simultaneously addressing the larger systemic barriers that limit successful smoke-free legislation.

While this review highlights barriers and mitigation strategies that are widely applicable, it also highlights the need for additional studies to explore and address barriers and opportunities related to social norms within specific cultures and contexts. These include not only smoking-specific norms (e.g. a pervasive ‘smoking culture’ in certain settings, social practices around cigarette sharing and gifting in China), but also broader norms impacting how individuals interact with one another more generally (e.g. avoiding confrontation)^27,34,101,113^. For example, one member of the National Tobacco Control Committee in the Gambia reported that ‘Maslaha Syndrome’ (socially accommodating negative habits or behaviors and trying to cover it up in order not to be blamed for reporting it) was a barrier to the success of smoke-free policies in the country^34^. Studies within our review highlight a need for educational efforts that address these social norms and emphasized the importance of engaging diverse stakeholders throughout policy development, implementation, enforcement, and evaluation, particularly stakeholders who may be most impacted by smoke-free policies (e.g. staff, business owners, people who smoke, law enforcement)^27,43,44,55,75,79^. In particular, participatory approaches can be useful for developing tailored, culturally appropriate policies and enforcement mechanisms for specific contexts^79^.

### Strengths and limitations

This review provides an overview of what is reported in the current literature related to smoke-free policies, including their development, implementation, enforcement, and compliance in each World Bank income strata and WHO region of the world. The study provides examples of key factors related to smoke-free policy development, implementation, enforcement, and compliance in different types of environments. This review also includes evidence from academic as well as gray literature and used consensus-based inclusion criteria.

Although this study used relevant databases and gray literature to produce a comprehensive literature search, only English articles were included in this study and only two databases were utilized in the search strategy. Therefore, it is possible that some literature may have been unintentionally excluded. Additionally, this study, building on previous work by Byron et al.^8^, excluded the 168 studies covered in that review, which reported findings from LMICs. Therefore, it is possible that the current study disproportionately reports findings from HICs. Despite these limitations, the results of this review provide useful insights to support international jurisdictions seeking to create 100% smoke-free environments through effective smoke-free policies.

This study followed the principles of a narrative review and excluded steps taken in other review processes such as conducting a risk of bias assessment and using a PICO framework for data extraction. However, a narrative review design allowed the research team to conduct a comprehensive overview of the global evidence on smoke-free policies given the large number of included articles and time-constraints of producing an up-to-date review. Moreover, this approach allowed the research team to answer a research question with wider parameters and put forward a more nuanced understanding of the complexities surrounding global smoke-free policies.

## CONCLUSION

Effective smoke-free policy development, implementation, compliance, and enforcement are invaluable to the establishment of 100% smoke-free environments. Drawing from these themes, this review highlights specific successful model policies in a variety of environments, identifies barriers to smoke-free legislation in LMICs and HICs, and provides insight into effective mitigation strategies at the global-level. The research presented in this study will support the establishment of 100% smoke-free environments, decrease SHS, and advance the objectives set forth by the FCTC.

## Data Availability

The data supporting this research are available from the authors on reasonable request.
