# Erratum: State of the literature discussing smoke-free policies globally: A narrative review

**DOI:** 10.18332/tid/178467

**Published:** 2024-01-19

**Authors:** European Publishing Production Team

**Affiliations:** 1 European Publishing, Heraklion, Greece

**Keywords:** tobacco, review, smoke-free policy, LMICs, HICs

**Erratum on:**

**State of the literature discussing smoke-free policies globally: A narrative review**

**By Jacqueline A. Teed, Meagan O. Robichaud, Michelle Duren, Hebe N. Gouda, Ryan David Kennedy**

**Tobacco Induced Diseases, Volume 22, Issue January, Page 1-17**

**Publish date: 5 January 2024**

**DOI: https://doi.org/10.18332/tid/174781**

In the corrected version of the article, a typesetting error regarding the name of the institution of the first three members of the authorship team was corrected from Johns Hopkings to Johns Hopkins. The mentioned changes are corrected also online.

