# Supplementary material for: State of the literature discussing smoke-free policies globally: A narrative review
Source: Tob Induc Dis. 2024 Jan 5;22:10.18332/tid/174781. doi: 10.18332/tid/174781 (PMC10767724; doi:10.18332/tid/174781)
Supplement: Supplementary file 1 [file TID-22-03-s1.pdf]

Search strategies from Pubmed included: (implement[tw] OR implementation[tw] OR implementing[tw] OR implemented[tw] OR enforce[tw] OR enforcement[tw] OR enforcing[tw] OR enforced[tw] OR comply[tw] OR compliance[tw] OR complying[tw] OR complied[tw] OR "guideline adherence"[MeSH Terms] OR "policy enforcement"[MeSH Terms]) AND (smoke-free[tw] OR smokefree[tw] OR "smoke free"[tw] OR ban[tw] OR bans[tw] OR banning[tw] OR banned[tw] OR restrict[tw] OR restriction[tw] OR restrictions[tw] OR restricting[tw] OR restricted[tw]) AND (("tobacco"[MeSH Terms] OR "tobacco products"[MeSH Terms]) OR tobacco[tw] OR "tobacco products"[MeSH Terms] OR "smoking"[MeSH Terms] OR smoking[tw] OR "smoke"[MeSH Terms] OR smoke[tw])

Search strategies for Embase included: (implement OR implementation OR implementing OR implemented OR enforce OR enforcement OR enforcing OR enforced OR comply OR compliance OR complying OR complied OR "guideline adherence" OR “policy enforcement”) AND (smoke-free OR smokefree OR "smoke free" OR ban OR bans OR banning OR banned OR restrict OR restriction OR restrictions OR restricting OR restricted) AND (tobacco OR tobacco OR "tobacco products" OR smoking OR smoking OR smoke OR smoke).

Description of Included Studies

First Author, Year	Country(ies)	Income Classification(s)	Region(s)	Environment(s)	Main Theme(s)	Citation
**Abdullah et al., 2022**	Bangladesh	Lower-middle income	SEARO	Unspecified	Development; Implementation	Abdullah SM, Wagner-Rizvi T, Huque R, Kanan S, Huque S, Ralston R, Collin J. 'A contradiction between our state and the tobacco company': conflicts of interest and institutional constraints as barriers to implementing Article 5.3 in Bangladesh. Tob Control. 2022; 31:s33–s38. doi:10.1136/tobaccocontrol-2021-057142
**Adams Jones et al., 2007**	United Kingdom: Wales	High-income	EURO	Indoor public places; Indoor workplaces	Implementation; Compliance; Enforcement	Adams Jones M, Adams Jones D, Powell C. Towards reality and away from blind acceptance: Changes in the attitude of public house bar staff to their exposure to second hand tobacco smoke in the work-place since the announcement of legislative measures. Health Educ J. 2007;66(4):323–34. doi:10.1177/0017896907083154
**Ahrens et al., 2005**	United States	High-income	AMRO	Indoor public places; Indoor workplaces	Implementation	Ahrens D, Uebelher P, Remington PL. Evaluation of community and organizational characteristics of smoke-free ordinance campaigns in 15 Wisconsin cities. Prev Chronic Dis. 2005;2(3):A08.
**Ahsan et al., 2022**	Pakistan	Lower-middle income	EMRO	Indoor public places; Indoor workplaces; Outdoor and quasi-outdoor environments	Implementation; Compliance; Enforcement	Ahsan H, Hoe C, Aslam F, Wright K, Cohen J, Kennedy R. Compliance with smoke-free policies at indoor and outdoor public places: an observational study in Pakistan. East Mediterr Health J. 2022;28(1):50–7. doi:10.26719/emhj.22.005

**Alpert et al., 2007**	United States	High-income	AMRO	Indoor public places; Indoor workplace	Compliance	Alpert HR, Carpenter CM, Travers MJ, Connolly GN. Environmental and economic evaluation of the Massachusetts smoke-free workplace law. J Community Health. 2007;32(4):269–81. doi:10.1007/s10900-007-9048-6
**Anderson et al., 2020**	Multiple: Global	Low-income; Lower-middle income; Upper-middle income; High-income	Multiple: Unspecified	Indoor public places; Indoor workplaces; Public transport	Development; Compliance; Enforcement	Anderson CL, Mons U, Winkler V. Global progress in tobacco control: the question of policy compliance. Glob Health Action. 2020;13(1):1844977. doi:10.1080/16549716.2020.1844977
**Antin et al., 2008**	United States	High-income	AMRO	Indoor workplaces	Implementation; Compliance; Enforcement	Antin TMJ, Moore RS, Lee JP, Satterlund TD. Law in Practice: Obstacles to a Smokefree Workplace Policy in Bars Serving Asian Patrons. J Immigr Minor Health. 2008;1–7. doi:10.1007/s10903-008-9174-y
**Antin et al, 2010**	United States	High-income	AMRO	Indoor workplaces	Implementation; Compliance; Enforcement	Antin TM, Moore RS, Lee JP, Satterlund TD. Law in practice: obstacles to a smokefree workplace policy in bars serving Asian patrons. J Immigr Minor Health Cent Minor Public Health. 2010;12(2):221–7. doi:10.1007/s10903-008-9174-y
**Anyanwu et al., 2020**	United Kingdom	High-income	EURO	Unspecified	Implementation; Compliance	Anyanwu PE, Craig P, Katikireddi SV, Green MJ. Impact of UK Tobacco Control Policies on Inequalities in Youth Smoking Uptake: A Natural Experiment Study. Nicotine Tob Res. 2020;22(11):1973–80. doi:10.1093/ntr/ntaa101

**Arifin et al., 2021**	Indonesia	Upper-middle income	SEARO	Indoor public places; Indoor workplaces	Implementation	Arifin MA, Nurhayani, Indar, Sirajuddin S, Susanti A, Ismaniar NI, et al. The implementationof non-smoking area policy in hospital of islam faisal makassar. Eur J Mol Clin Med. 2021;8(1):1358–65.
**Arora et al., 2012**	India	Lower-middle income	SEARO	Indoor public places; Indoor workplaces	Development; Implementation	Arora M, Mathur MR, Singh N. A Framework to Prevent and Control Tobacco among Adolescents and Children: Introducing the IMPACT Model. Indian Journal of Pediatrics. 2013;80(Suppl 1):S55-S62. doi:10.1007/s12098-012-0768-y
**Asyary et al., 2021**	Indonesia	Upper-middle income	SEARO	Indoor public places; Indoor workplaces	Implementation; Compliance	Asyary A, Veruswati M, Arianie CP, Ratih TSD, Hamzah A. Prevalence of Smoke-Free Zone Compliance among Schools in Indonesia: A Nationwide Representative Survey. Asian Pac J Cancer Prev APJCP. 2021;22(2):359–63. doi:10.31557/APJCP.2021.22.2.359
**Avila-Tang et al., 2010**	Multiple	Lower-middle income; Upper-middle income; High-income	AMRO	Indoor public places; Indoor workplaces	Development; Implementation; Compliance	Avila-Tang E, Travers MJ, Navas-Acien A. Promoting smoke-free environments in Latin America: A comparison of methods to assess secondhand smoke exposure. Salud Publica Mex. 2010;52(Suppl 2):S138–48. doi:10.1590/S0036-36342010000800009
**Awan et al., 2018**	Multiple	High-income	EMRO	Unspecified	Development; Implementation	Awan KH, Hussain QA, Khan S, Peeran SW, Hamam MK, Hadlaq EA, et al. Accomplishments and challenges in tobacco control endeavors - Report from the Gulf Cooperation Council countries. Saudi Dent J. 2018;30(1):13–8. doi:10.1016/j.sdentj.2017.08.003

**Bajoga et al., 2011**	Multiple	High-income	AMRO, EURO, WPRO	Indoor public places; Indoor workplaces	Development; Implementation	Bajoga U, Lewis S, McNeill A & Szatkowski L. Does the introduction of comprehensive smoke-free legislation lead to a decrease in population smoking prevalence? Addiction. 2011;106(7):1346-54. doi:10.1111/j.1360-0443.2011.03446.x
**Ballbè et al., 2012**	Spain	High-income	EURO	Indoor public places; Indoor workplaces; Outdoor and quasi-outdoor environments	Implementation; Compliance	Ballbè M, Nieva G, Mondon S, Pinet C, Bruguera E, Saltó E, Fernández E, Gual A, and the Smoking and Mental Health Group. Smoke-free policies in psychiatric services: identification of unmet needs. Tobacco Control. 2012; 21(5): 549-54. doi:10.1136/tobaccocontrol-2011-050029
**Balwicki et al., 2016**	Poland	High-income	EURO	Unspecified	Development; Implementation	Balwicki Ł, Stokłosa M, Balwicka-Szczyrba M, Tomczak W. Tobacco industry interference with tobacco control policies in Poland: legal aspects and industry practices. Tob Control. 2016;25(5):521–6. doi:10.1136/tobaccocontrol-2015-052582
**Bardach et al., 2020**	Multiple	Lower-middle income; Upper-middle income; High-income	AMRO	Unspecified	Development; Implementation; Compliance; Enforcement	Bardach A, Rodríguez MB, Ciapponi A, Augustovsky F, Alcaraz A, Soto N, Virgilio S, Reynales Shigematsu LM, Roberti J, Pichón-Riviere A. Smoke-free air interventions in Seven Latin American Countries: Health and Financial Impact to Inform Evidence-Based Policy Implementation. Nicotine & Tobacco Research. 2020; 22(12):2149-57. doi:10.1093/ntr/ntaa133
**Barry et al., 2022**	Multiple	Low-income; Lower-middle income	AFRO, SEARO	Unspecified	Development; Implementation; Compliance	Barry RA, Mackintosh AM, Zhao L, Hiilamo H. Challenges and opportunities in implementing Article 5.3 of the WHO Framework Convention on Tobacco Control: A qualitative analysis of experiences across countries. Tobacco Control. 2022;31(Suppl 2):s46-s52. doi:10.1136/tobaccocontrol-2021-057154

**Basnet et al., 2022**	Nepal	Lower-middle income	SEARO	Indoor public places; Indoor workplaces; Outdoor and quasi-outdoor environments; Public transport	Implementation; Compliance; Enforcement	Basnet LB, Budhathoki SS, Adhikari B, Thapa J, Neupane B, Moses T, et al. Compliance with the smoke-free public places legislation in Nepal: A cross-sectional study from Biratnagar Metropolitan City. PLoS ONE. 2022;17(3):e0264895. doi:10.1371/journal.pone.0264895
**Bhatta et al., 2020**	Nepal	Lower-middle income	SEARO	Indoor public places; Indoor workplaces; Outdoor and quasi-outdoor environments	Development; Implementation	Bhatta DN, Crosbie E, Bialous SA, Glantz S. Defending Comprehensive Tobacco Control Policy Implementation in Nepal From Tobacco Industry Interference (2011-2018). Nicotine Tob Res. 2020;22(12):2203–12. doi: 10.1093/ntr/ntaa067
**Bhatta et al., 2020**	Nepal	Lower-middle income	SEARO	Indoor public places; Indoor workplaces; Outdoor and quasi-outdoor environments	Development; Implementation; Enforcement	Bhatta DN, Bialous S, Crosbie E, Glantz S. Exceeding WHO framework Convention on Tobacco Control (FCTC) obligations: Nepal overcoming tobacco industry interference to enact a comprehensive tobacco control policy. Nicotine Tob Res. 2020;22(12):2213–23. doi:10.1093/ntr/ntz177
**Boes et al., 2015**	Switzerland	High-income	EURO	Indoor public places; Indoor workplaces	Compliance; Enforcement	Boes S, Marti J, MacLean JC. The Impact of Smoking Bans on Smoking and Consumer Behavior: Quasi-Experimental Evidence from Switzerland. Health Econ U K. 2015;24(11):1502–16. doi: 10.1002/hec.3108
**Bogdanovica et al., 2011**	Multiple	High-income	EURO	Indoor public places; Indoor workplaces; Public transport	Development; Implementation; Compliance; Enforcement	Bogdanovica I, McNeill A, Murray R, Britton J. What Factors Influence Smoking Prevalence and Smoke Free Policy Enactment across the European Union Member States. PLoS ONE. 2011;6(8):e23889. doi:10.1371/journal.pone.0023889

**Borland et al., 2006**	Multiple	High-income	AMRO, EURO, WPRO	Indoor public places; Indoor workplaces	Implementation; Compliance; Enforcement	Borland R, Yong HH, Siahpush M, Hyland A, Campbell S, Hastings G, et al. Support for and reported compliance with smoke-free restaurants and bars by smokers in four countries: Findings from the International Tobacco Control (ITC) Four Country Survey. Tob Control. 2006;15(SUPPL. 3):iii34–41. doi:10.1136/tc.2004.008748
**Bosdriesz et al., 2015**	Multiple	High-income	EURO	Indoor public places; Indoor workplaces	Development; Implementation	Bosdriesz JR, Willemsen MC, Stronks K, Kunst AE. Tobacco control policy development in the European Union: do political factors matter? Eur J Public Health. 2015;25(2):190–4. doi:10.1093/eurpub/cku197
**Brathwaite et al., 2015**	Multiple	Low-income; Lower-middle income; Upper-middle income	AFRO	Indoor public places	Development; Implementation	Brathwaite R, Addo J, Smeeth L, Lock K. A Systematic Review of Tobacco Smoking Prevalence and Description of Tobacco Control Strategies in Sub-Saharan African Countries; 2007 to 2014. PLoS ONE. 2015;10(7): 1–16. doi:10.1371/journal.pone.0132401
**Buettner-Schmidt et al., 2017**	United States	High-income	AMRO	Indoor public places; Outdoor and quasi-outdoor environments	Compliance; Enforcement	Buettner-Schmidt K, Miller DR. An observational study of compliance with North Dakota's smoke-free law among retail stores that sell electronic smoking devices. Tob Control. 2017;26(4):452–4. doi:10.1136/tobaccocontrol-2015-052888
**Byron et al., 2016**	Indonesia	Upper-middle income	SEARO	Indoor public places; Indoor workplaces; Outdoor and quasi-outdoor environments; Public transport	Implementation; Compliance; Enforcement	Byron MJ, Cohen JE, Frattaroli S, Gittelsohn J, Jernigan DH. Using the theory of normative social behavior to understand compliance with a smoke-free law in a middle-income country. Health Educ Res. 2016;31(6):738–48. doi:10.1093/her/cyw043

**Byron et al., 2018**	Multiple: Global	Lower-middle income; Upper-middle	AFRO, AMRO, EMRO, EURO, SEARO, WPRO	Indoor public places; Indoor workplaces	Implementation; Compliance; Enforcement	Byron MJ, Cohen J, Frattaroli S, Gittelsohn J, Jernigan D. Implementing smoke-free laws in low and middle-income countries: A systematic literature review and proposed research Agenda. Tob Induc Dis. 2018;16:274. doi:10.18332/tid/110007
**Byron et al., 2019**	Multiple: Global	Lower-middle income; Upper-middle	AFRO, AMRO, EMRO, EURO, SEARO, WPRO	Indoor public places; Indoor workplaces	Implementation; Compliance; Enforcement	Byron MJ, Cohen JE, Frattaroli S, Gittelsohn J, Drope JM, Jernigan DH. Implementing smoke-free policies in low- and middle-income countries: A brief review and research agenda. Tobacco Induced Diseases. 2019;17(August):60. doi:10.18332/tid/110007
**Calo et al., 2013**	United States	High-income	AMRO	Indoor public places; Indoor workplaces; Outdoor and quasi-outdoor environments	Development; Implementation; Compliance	Calo WA, Krasny SE. Environmental determinants of smoking behaviors: The role of policy and environmental interventions in preventing smoking initiation and supporting cessation. Curr Cardiovasc Risk Rep. 2013;7(6):446–52. doi:10.1007/s12170-013-0344-7
**Chaaya et al., 2013**	Lebanon	Lower-middle income	EMRO	Indoor public places; Indoor workplaces	Implementation; Compliance; Enforcement	Chaaya M, Alameddine M, Nakkash R, Afifi RA, Khalil J, Nahhas G. Students' attitude and smoking behaviour following the implementation of a university smoke-free policy: a cross-sectional study. BMJ Open. 2013;3:e002100. doi:10.1136/bmjopen-2012-002100
**Cham et al., 2021**	The Gambia	Low-income	AFRO	Indoor public places; Outdoor and quasi-outdoor environments	Implementation; Compliance	Cham B, Mdege ND, Bauld L, Britton J, D'Alessandro U. Exposure to Second-Hand Smoke in Public Places and Barriers to the Implementation of Smoke-Free Regulations in The Gambia: A Population-Based Survey. Int. J. Environ. Res. Public Health. 2021;18(12):6263. doi:10.3390/ijerph18126263

**Champion et al., 2005**	Australia	High-income	WPRO	Indoor public places; Indoor workplaces	Development; Implementation	Champion D, Chapman S. Framing pub smoking bans: An analysis of Australian print news media coverage, March 1996-March 2003. J Epidemiol Community Health. 2005;59(8):679–84. doi:10.1136/jech.2005.035915
**Charoenca et al., 2012**	Thailand	Upper-middle income	SEARO	Unspecified	Development; Implementation	Charoenca N, Mock J, Kungskulniti N, Preechawong S, Kojetin N, Hamann SL. Success counteracting tobacco company interference in Thailand: An example of FCTC implementation for low- and middle-income countries. Int J Environ Res Public Health. 2012;9(4):1111–34. doi:10.3390/ijerph9041111
**Charoenca et al., 2021**	Thailand	Upper-middle income	SEARO	Indoor public places; Indoor workplaces; Outdoor and quasi-outdoor environments; Public transport	Development; Implementation; Compliance; Enforcement	Charoenca N, Kungskulniti N, Pipattanachat V, Pitayarangsarit S, Hamann S, Mock J. The Implementation Activist: How One Determined Person with a Camera Has Achieved Enforcement of Smoke-Free Laws throughout Thailand. Asian Pac J Cancer Prev APJCP. 2021;22(S2):19–34. doi:10.31557/APJCP.2021.22.S2.19
**Chatterjee et al., 2017**	India	Lower-middle income	SEARO	Indoor public places; Indoor workplaces	Development; Implementation; Compliance; Enforcement	Chatterjee N, Kadam R, Patil D, Todankar P. Adherence to the Tobacco-Free School Policy in Rural India. Asian Pac J Cancer Prev APJCP. 2017;18(9):2367–73. doi:10.22034/APJCP.2017.18.9.2367
**Chaudhary et al., 2019**	India	Lower-middle income	SEARO	Indoor public places	Implementation; Compliance	Chaudhary A, Thakur A, Chauhan T, Mahajan A, Barwal VK, Chamotra S, et al. Creation of a Smoke-free Environment for Children: An Assessment of Compliance to COTPA 2003 Legislation in an Urban Area. Indian Pediatr. 2019;56(9):837–40. doi:10.1007/s13312-019-1609-0

**Chung-Hall et al., 2019**	Multiple: Global	Low-income; Lower-middle income; Upper-middle income; High-income	AFRO, AMRO, EURO, SEARO, WPRO	Indoor public places; Indoor workplaces; Outdoor and quasi-outdoor environments; Public transport	Development; Implementation; Enforcement	Chung-Hall J, Craig L, Gravely S, Sansone N, Fong GT. Impact of the WHO FCTC over the first decade: a global evidence review prepared for the Impact Assessment Expert Group. Tob Control. 2019;28:s119–28. doi:10.1136/tobaccocontrol-2018-054389
**Chyderiotis et al., 2019**	France	High-income	EURO	Indoor public places; Indoor workplaces	Implementation; Compliance	Chyderiotis S, Beck F, Andler R, Hitchman SC, Benmarhnia T. How to reduce biases coming from a before and after design: the impact of the 2007-08 French smoking ban policy. Eur J Public Health. 2019;29(2):372–7. doi:10.1093/eurpub/cky160
**Cormac et al., 2008**	United Kingdom & Ireland	High-income	EURO	Indoor public places; Indoor workplaces; Outdoor and quasi-outdoor environments	Development; Implementation	Cormac I, McNally L. How to implement a smoke-free policy. Adv Psychiatr Treat. 2008;14(3):198–207. doi:10.1192/apt.bp.107.004069.
**Croghan et al., 2011**	Italy	High-income	EURO	Indoor public places; Indoor workplaces	Development; Implementation	Croghan I, Muggli M, Zagà V, Lockhart N, Ebbert J, Mangiaracina G, et al. Lessons learned on the road to a smoke-free Italy. Ann Ig Med Prev E Comunita. 2011;23(2):125–36. PMID: 21770229
**Dean et al., 2018**	Australia	High-income	WPRO	Indoor public places; Indoor workplaces	Implementation	Dean TD, Cross W, Munro I. An Exploration of the Perspectives of Associate Nurse Unit Managers Regarding the Implementation of Smoke-free Policies in Adult Mental Health Inpatient Units. Issues Ment Health Nurs. 2018;39(4):328–36. doi:10.1080/01612840.2017.1413461

**Donchin et al., 2004**	Israel	High-income	EURO	Indoor public places; Indoor workplaces; Outdoor and quasi-outdoor environments	Development; Implementation; Compliance; Enforcement	Donchin M, Baras M. A "smoke-free" hospital in Israel--a possible mission. Prev Med. 2004;39(3):589–95. doi:10.1016/j.ypmed.2004.02.020
**Edwards et al., 2008**	New Zealand	High-income	WPRO	Indoor public places; Indoor workplaces	Compliance; Enforcement	Edwards R, Thomson G, Wilson N, Waa A, Bullen C, O'Dea D, et al. After the smoke has cleared: evaluation of the impact of a new national smoke-free law in New Zealand. Tob Control. 2008;17(1):e2. doi:10.1136/tc.2007.020347
**Edwards et al., 2009**	New Zealand	High-income	WPRO	Indoor workplaces	Implementation	Edwards R, Gifford H, Waa A, Glover M, Thomson G, Wilson N. Beneficial impacts of a national smokefree environments law on an indigenous population: a multifaceted evaluation. International Journal for Equity in Health. 2009;8:12. doi:10.1186/1475-9276-8-12
**El Amin et al., 2019**	Sudan	Low-income	AFRO	Indoor public places; Indoor workplaces	Compliance; Enforcement	El Amin SET. School Smoking Policies and Health Science Students' Use of Cigarettes, Shisha, and Dipping Tombak in Sudan. Front Public Health. 2019;7:290. doi:10.3389/fpubh.2019.00290
**El-Awa et al., 2020**	Multiple	Low-income; Lower-middle income; Upper-middle income; High-income	EMRO	Indoor public places; Outdoor and quasi-outdoor environments	Implementation	El-Awa F, Bettcher D, Al-Lawati JA, Alebshehy R, Gouda H, Fraser CP. The status of tobacco control in the eastern mediterranean region: Progress in the implementation of the MPOWER measures. East Mediterr Health J. 2020;26(1):102–9. doi:10.26719/2020.26.1.102

**Fallin et al., 2014**	United States	High-income	AMRO	Indoor public places	Development; Implementation	Fallin A, Goodin A, Rayens MK, Morris S, Hahn EJ. Smoke-free policy implementation: theoretical and practical considerations. Policy Polit Nurs Pract. 2014;15(3–4):81–92. doi:10.1177/1527154414562301
**Fallin-Bennett et al., 2017**	United States	High-income	AMRO	Indoor public places; Outdoor and quasi-outdoor environments	Development; Implementation; Compliance; Enforcement	Fallin-Bennett A, Roditis M, Glantz SA. The carrot and the stick? Strategies to improve compliance with college campus tobacco policies. J Am Coll Health J ACH. 2017;65(2):122–30. doi:10.1080/07448481.2016.1262380
**Farrelly et al., 2017**	United States	High-income	AMRO	Indoor public places; Indoor workplaces; Public transport	Development; Implementation	Farrelly MC, Chaloupka FJ, Berg CJ, Emery SL, Henriksen L, Ling P, et al. Taking Stock of Tobacco Control Program and Policy Science and Impact in the United States. J Addict Behav Ther. 2017;1(2). PMID: 30198028
**Fong et al., 2013**	France	High-income	EURO	Indoor public places; Indoor workplaces; Outdoor and quasi-outdoor environments	Implementation; Compliance	Fong GT, Craig LV, Guignard R, Nagelhout GE, Tait MK, Driezen P, et al. Evaluating the Effectiveness of France's Indoor Smoke-Free Law 1 Year and 5 Years after Implementation: Findings from the ITC France Survey. PLoS One. 2013;8(6):e66692. doi:10.1371/journal.pone.0066692
**Freiburghaus et al., 2021**	Multiple	High-income	EURO	Indoor public places; Indoor workplaces; Outdoor and quasi-outdoor environments	Implementation; Enforcement	Freiburghaus T, Raffing R, Ballbè M, Gual A, Tönnesen H. The right to smoke and the right to smoke-free surroundings: international comparison of smokefree psychiatric clinic implementation experiences. BJPsych Open. 2021;7:e81, 1–6. doi:10.1192/bjo.2021.35

**Garritsen et al., 2021**	Netherlands	High-income	EURO	Outdoor and quasi-outdoor environments	Implementation; Compliance; Enforcement	Garritsen HH, Rozema AD, van de Goor IAM, Kunst AE. Smoke-free sports in the netherlands: Why most sports clubs have not adopted an outdoor smoke-free policy. Int J Environ Res Public Health. 2021;18(5):1–9. doi:10.3390/ijerph18052454
**Garritsen et al., 2021**	Netherlands	High-income	EURO	Outdoor and quasi-outdoor environments	Development; Implementation; Compliance; Enforcement	Garritsen HH, Rozema AD, van de Goor I, Kunst AE. Implementation of an outdoor smoke-free policy at sports clubs: Critical situations and determinants influencing implementation. International Journal of Drug Policy. 2021;92:[103129]. doi:10.1016/j.drugpo.2021.103129
**Gatto et al., 2019**	United States	High-income	AMRO	Indoor public places; Indoor workplaces	Implementation; Compliance; Enforcement	Gatto A, Powell SE, Walters EF, Zamani S, Sales LB, DeBate R. A Mixed-Methods Assessment of a Peer-Enforced Tobacco- and Smoke-Free Policy at a Large Urban University. J Community Health. 2019;44(2):365–76. doi:10.1007/s10900-018-0593-y
**Geiger et al., 2012**	United States	High-income	AMRO	Indoor public places; Indoor workplaces	Development; Implementation; Enforcement	Geiger BF, Vaid I, Beeson D, Riddle B. Implementation of School Policies to Prevent Youth Tobacco Use in Alabama. J Sch Health. 2012;82(6):277–84. doi:10.1111/j.1746-1561.2012.00698.x
**Goel et al., 2018**	India	Lower-middle income	SEARO	Indoor public places; Indoor workplaces; Outdoor and quasi-outdoor environments; Public transport	Development; Implementation; Compliance; Enforcement	Goel S, Sharma D, Gupta R, Mahajan V. Compliance with smoke-free legislation and smoking behaviour: observational field study from Punjab, India. Tob Control. 2018;27(4):407–13. doi:10.1136/tobaccocontrol-2016-053559

**Gonzalez et al., 2013**	Netherlands	High-income	EURO	Indoor public places; Indoor workplaces	Development; Implementation	Gonzalez M, Glantz SA. Failure of policy regarding smoke-free bars in the Netherlands. Eur J Public Health. 2013;23(1):139–45. doi:10.1093/eurpub/ckr173
**Gorini et al., 2011**	Italy	High-income	EURO	Indoor public places; Indoor workplaces; Outdoor and quasi-outdoor environments; Public transport	Development; Implementation	Gorini G, Currie L, Spizzichino L, Galeone D, Lopez MJ. Smoke-free policy development in Italy through the legislative process of the ban 2000-2005, and press media review 1998-2008. Ann Ist Super Sanita. 2011;47(3):260–5. doi:10.4415/ANN_11_03_04
**Grant et al., 2014**	United States	High-income	AMRO	Indoor public places; Indoor workplaces	Implementation; Compliance; Enforcement	Grant LG, Oliffe JL, Johnson JL, Bottorff JL. Health care professionals implementing a smoke-free policy at inpatient psychiatric units. Qual Health Res. 2014;24(12):1732–44. doi:10.1177/1049732314549026
**Gravely et al., 2018**	Uganda	Low-income	AFRO	Indoor public places; Indoor workplaces; Outdoor and quasi-outdoor environments	Implementation; Compliance; Enforcement	Gravely S, Nyamurungi KN, Kabwama SN, Okello G, Robertson L, Heng KK, Ndikum AE, Oginni AS, Rusatira JC, Kakoulides S, Huffman MD, Yusuf S, Bianco E. Knowledge, opinions and compliance related to the 100% smoke-free law in hospitality venues in Kampala, Uganda: cross-sectional results from the KOMPLY Project. BMJ Open. 2018;8:e017601. doi:10.1136/bmjopen-2017-017601
**Halkett et al., 2010**	New Zealand	High-income	WPRO	Outdoor and quasi-outdoor environments	Development; Implementation	Halkett L, Thomson G. Getting an outdoor smokefree policy: The case of Kapiti Coast District Council. N Z Med J. 2010;123(1308):28–40. PMID:20173803

**Harizi et al., 2020**	Tunisia	Lower-middle income	EMRO	Indoor public places; Indoor workplaces; Public transport	Implementation; Compliance; Enforcement	Harizi C, El-Awa F, Ghedira H, Audera-Lopez C, Fakhfakh R. Implementation of the WHO Framework Convention on Tobacco Control in Tunisia: Progress and challenges. Tob Prev Cessat. 2020;6(December):72. doi:10.18332/tpc/130476
**Harris et al., 2009**	United States	High-income	AMRO	Outdoor and quasi-outdoor environments	Compliance; Enforcement	Harris KJ, Stearns JN, Kovach RG, Harrar SW. Enforcing an outdoor smoking ban on a college campus: Effects of a multicomponent approach. J Am Coll Health. 2009;58(2):121–6. doi:10.1080/07448480903221285
**Haw et al., 2006**	United Kingdom: Scotland	High-income	EURO	Indoor public places; Indoor workplaces	Implementation	Haw SJ, Gruer L, Amos A, Currie C, Fischbacher C, Fong GT, et al. Legislation on smoking in enclosed public places in Scotland: How will we evaluate the impact? J Public Health. 2006;28(1):24–30. doi:10.1093/pubmed/fdi080
**Heydari et al., 2018**	Multiple	Low-income; Lower-middle income; Upper-middle income; High-income	EMRO	Unspecified	Development; Implementation	Heydari G, Zaatari G, Al-Lawati JA, El-Awa F, Fouad H. Mpower, needs and challenges: Trends in the implementation of the who fctc in the eastern mediterranean region. East Mediterr Health J. 2018;24(1):63–71. doi:10.26719/2018.24.1.63
**Heydari et al., 2020**	Multiple	Low-income; Lower-middle income; Upper-middle income; High-income	EMRO	Unspecified	Implementation; Compliance	Heydari G. A decade after introducing MPOWER, trend analysis of implementation of the WHO FCTC in the Eastern Mediterranean Region. Lung India. 2020;37(2):120–5. doi:10.4103/lungindia.lungindia_388_19

**Hoffman et al., 2020**	Multiple	High-income	EURO	Indoor public places; Indoor workplaces	Development; Implementation	Hoffmann L, Mlinarić M, Mï Lard N, Leï OT, Grard A, Lindfors P, et al. "[…] the situation in the schools still remains the Achilles heel." Barriers to the implementation of school tobacco policies-a qualitative study from local stakeholder's perspective in seven European cities. Health Educ Res. 2020;35(1):32–43. doi:10.1093/her/cyz037
**Jallow et al., 2019**	The Gambia	Low-income	AFRO	Indoor public places; Indoor workplaces; Public transport	Development; Implementation	Jallow IK, Britton J, Langley T. Exploration of Policy Makers' Views on the Implementation of the Framework Convention on Tobacco Control in the Gambia: A Qualitative Study. Nicotine Tob Res. 2019;21(12):1652–9. doi:10.1093/ntr/ntz003
**Kennedy et al., 2014**	Canada	High-income	AMRO	Outdoor and quasi-outdoor environments	Enforcement	Kennedy RD, Zummach D, Filsinger S, Leatherdale ST. Reported municipal costs from outdoor smokefree by-laws-experience from Ontario, Canada. Tobacco Induced Diseases. 2014;12(1):4. doi:10.1186/1617-9625-12-4
**Khan et al., 2019**	Bangladesh	Lower-middle income	SEARO	Indoor public places; Public transport	Development; Implementation	Khan MK, Hoque HE, Ferdous J. Knowledge and Attitude Regarding National Tobacco Control Law and Practice of Tobacco Smoking among Bangladesh Police. Mymensingh Med J. 2019;28(4):752–61. PMID:31599237
**Králíková et al., 2020**	Czech Republic	High-income	EURO	Indoor workplaces	Implementation	Králíková E, Pánková A. Barriers to introduction of smoke-free workplaces in Central Europe: example of the Czech Republic. Cent Eur J Public Health. 2020;28:S22–5. doi:10.21101/cejph.a6160

**Kumar et al., 2022**	India	Lower-middle income	SEARO	Unspecified	Development; Implementation	Kumar P, Barry RA, Kulkarni MM, Kamath VG, Ralston R, Collin J. Institutional tensions, corporate social responsibility and district-level governance of tobacco industry interference: analysing challenges in local implementation of Article 5.3 measures in Karnataka, India. Tob Control. 2022;31(suppl 2):s26–s32. doi:10.1136/tobaccocontrol-2021-057113
**Kungskulniti et al., 2018**	Thailand	Upper-middle income	SEARO	Indoor public places; Indoor workplaces; Public transport	Development; Implementation; Compliance; Enforcement	Kungskulniti N, Pitayarangsarit S, Hamann SL. Stakeholder's Assessment of the Awareness and Effectiveness of Smoke-free Law in Thailand. Int J Health Policy Manag. 2018;7(10):919–22. doi:10.15171/ijhpm.2018.47
**Kunyk et al., 2007**	Canada	High-income	AMRO	Indoor public places; Indoor workplaces; Outdoor and quasi-outdoor environments	Development; Implementation; Compliance; Enforcement	Kunyk D, Els C, Predy G, Haase M. Development and introduction of a comprehensive tobacco control policy in a Canadian regional health authority. Prev Chronic Dis. 2007;4(2):A30.
**Kvillemo et al., 2021**	Sweden	High-income	EURO	Outdoor and quasi-outdoor environments	Implementation; Compliance; Enforcement	Kvillemo P, Feltmann K, Elgán TH, Gripenberg J. Evaluation of the Implementation of a 25-Year Outdoor School Ground Smoking Ban: A Qualitative Interview Study With Implications for Prevention Practise. Front Public Health. 2021;9:628748. doi:10.3389/fpubh.2021.628748
**Laird et al., 2019**	United Kingdom: Scotland	High-income	EURO	Unspecified	Development; Implementation	Laird Y, Myers F, Reid G, McAteer J. Tobacco Control Policy in Scotland: A Qualitative Study of Expert Views on Successes, Challenges and Future Actions. International Journal of Environmental Research and Public Health. 2019; 16(15):2659. doi:10.3390/ijerph16152659

**Lawn et al., 2010**	Australia	High-income	WPRO	Indoor public places; Indoor workplaces	Implementation; Enforcement; Compliance	Lawn S, Campion J. Factors associated with success of smoke-free initiatives in Australian psychiatric inpatient units. Psychiatr Serv. 2010;61(3):300–5. doi:10.1176/ps.2010.61.3.300
**Lawn et al., 2013**	Multiple	Upper-middle income; High-income	AMRO, EURO, WPRO	Indoor public places; Indoor workplaces	Implementation	Lawn S, Campion J. Achieving smoke-free mental health services: Lessons from the past decade of implementation research. Int J Environ Res Public Health. 2013;10(9):4224–44. doi:10.3390/ijerph10094224
**Lawn et al., 2015**	United Kingdom: England	High-income	EURO	Indoor public places; Indoor workplaces	Implementation; Compliance; Enforcement	Lawn S, Feng Y, Tsourtos G, Campion J. Mental health professionals' perspectives on the implementation of smoke-free policies in psychiatric units across England. Int J Soc Psychiatry. 2015;61(5):465–74. doi:10.1177/0020764014553002
**Leung et al., 2013**	United States	High-income	AMRO	Outdoor and quasi-outdoor environments	Development; Implementation	Leung R, Mallya G, Dean LT, Rizvi A, Dignam L, Schwarz DF. Instituting a smoke-free policy for city recreation centers and playgrounds, Philadelphia, Pennsylvania, 2010. Prev Chronic Dis. 2013;10:E116. doi:10.5888/pcd10.120294
**Lidegaard et al., 2021**	Denmark	High-income	EURO	Indoor workplaces	Implementation; Compliance; Enforcement	Lidegaard LP, Kristiansen M, Pisinger C. Readiness for implementation of smoke-free work hours in private companies: A qualitative study of perceptions among middle managers. Tob Prev Cessat. 2021;7:38. doi:10.18332/tpc/134800

**Lin et al., 2019**	China	Upper-middle income	WPRO	Indoor public places; Indoor workplaces; Public transport	Implementation; Compliance; Enforcement	Lin H, Chang C, Liu Z, Zheng Y. Subnational smoke-free laws in China. Tobacco Induced Diseases. 2019;17(November):78. doi:10.18332/tid/112665
**Lock et al., 2010**	United Kingdom: England	High-income	EURO	Indoor public places	Implementation; Compliance; Enforcement	Lock K, Adams E, Pilkington P, Duckett K, Gilmore A, Marston C. Evaluating social and behavioural impacts of english smoke-free legislation in different ethnic and age groups: Implications for reducing smoking-related health inequalities. Tob Control. 2010;19(5):391–7. doi:10.1136/tc.2009.032318
**Martinez et al., 2009**	Spain	High-income	EURO	Indoor public places; Indoor workplaces	Development; Implementation	Martinez C. Barriers and challenges of implementing tobacco control policies in hospitals: applying the institutional analysis and development framework to the Catalan Network of Smoke-Free Hospitals. Policy Polit Nurs Pract. 2009;10(3):224–32. doi:10.1177/1527154409346736
**Mele et al., 2010**	Italy	High-income	EURO	Indoor public places; Indoor workplaces; Public transport	Development; Implementation	Mele V, Compagni A. Explaining the unexpected success of the smoking ban in Italy: political strategy and transition to practice, 2000–2005. Public Adm. 2010;88(3):819–35. doi:10.1111/j.1467-9299.2010.01840.x
**Miller at al., 2007**	Australia	High-income	WPRO	Indoor public places; Indoor workplaces	Development; Implementation; Compliance; Enforcement	Miller CL, Hickling JA. Phased in smoke-free workplace laws: impact in grass-roots pubs and clubs in South Australia. Health Promot J Aust Off J Aust Assoc Health Promot Prof. 2007;18(1):26–32. doi:10.1071/HE07026

**Mlinarić et al., 2019**	Multiple	High-income	EURO	Indoor public places	Development; Implementation; Compliance	Mlinarić M, Hoffmann L, Kunst AE, Schreuders M, Willemsen MC, Moor I, et al. Explaining Mechanisms That Influence Smoke-Free Implementation at the Local Level: A Realist Review of Smoking Bans. Nicotine Tob Res. 2019;21(12):1609–20. doi:10.1093/ntr/nty206
**Mohan et al., 2013**	India	Lower-middle income	SEARO	Unspecified	Implementation; Compliance; Enforcement	Mohan S, Mini GK, Thankappan KR. High knowledge of Framework Convention on Tobacco Control provisions among local government representatives does not translate into effective implementation: Findings from Kerala, India. Public Health. 2013;127(2):178–81. doi:10.1016/j.puhe.2012.11.018
**Mons et al., 2012**	Multiple	High-income	EURO	Indoor public places; Indoor workplaces	Implementation	Mons U, Nagelhout GE, Guignard R, McNeill A, van den Putte B, Willemsen MC, et al. Comprehensive smoke-free policies attract more support from smokers in Europe than partial policies. Eur J Public Health. 2012;22 Suppl 1:10–6. doi:10.1093/eurpub/ckr202
**Montini et al., 2008**	United States	High-income	AMRO	Indoor public places; Indoor workplaces	Implementation; Compliance; Enforcement	Montini T, Bero LA. Implementation of a workplace smoking ban in bars: the limits of local discretion. BMC Public Health. 2008;8:402. doi:10.1186/1471-2458-8-402
**Moreland-Russell et al., 2015**	United States	High-income	AMRO	Unspecified	Development; Implementation	Moreland-Russell S, Carothers BJ. An examination of two policy networks involved in advancing smokefree policy initiatives. Int J Environ Res Public Health. 2015;12(9):11117–31. doi:10.3390/ijerph120911117

**Nagelhout et al., 2011**	Multiple	High-income	EURO	Indoor public places; Indoor workplaces	Implementation; Compliance; Enforcement	Nagelhout GE, Mons U, Allwright S, Guignard R, Beck F, Fong GT, et al. Prevalence and predictors of smoking in "smoke-free" bars. Findings from the International Tobacco Control (ITC) Europe Surveys. Soc Sci Med. 2011;72(10):1643–51. doi:10.1016/j.socscimed.2011.03.018
**Nguyen et al., 2020**	Vietnam	Lower-middle income	WPRO	Indoor public places; Indoor workplaces	Implementation; Compliance	Nguyen NB, Capra M, Johnstone K, Vu HL, Tran V, Nguyen MT, et al. Change in Compliance of Staff at 4 Vietnam Universities after the Enactment of Smoke-free Environment Decree. Env Health Insights. 2020;14:1178630220972957. doi:10.1177/1178630220972
**Peruga et al., 2017**	Multiple	Low-income; Lower-middle income; Upper-middle income; High-income	AFRO, AMRO, EMRO, EURO, SEARO, WPRO	Indoor public places; Indoor workplaces; Public transport	Implementation; Compliance; Enforcement	Peruga A, Hayes LS, Aguilera X, et al. Correlates of compliance with national comprehensive smoke-free laws. Tobacco Control. 2018;27:608-13. doi:10.1136/tobaccocontrol-2017-053920
**Peruga et al., 2021**	Chile	High-income	AMRO	Indoor public places; Indoor workplaces; Outdoor and quasi-outdoor environments	Compliance; Enforcement	Peruga A, Molina X, Delgado I, Matute I, Olea A, Hirmas M, et al. Compliance with the smoking ban in enclosed, semiopen and open areas of workplaces and public places in Chile. Tob Control. 2021;30(5):570–3. doi:10.1136/ tobaccocontrol-2020-055632
**Radwan et al., 2012**	Egypt	Lower-middle income	EMRO	Indoor public places; Indoor workplaces	Development; Implementation; Enforcement	Radwan GN, Loffredo CA, Aziz R, Abdel-Aziz N, Labib N. Implementation, barriers and challenges of smoke-free policies in hospitals in Egypt. BMC Res Notes. 2012;5:568. doi:10.1186/1756-0500-5-568

**Ramachandran et al., 2020**	United States	High-income	AMRO	Indoor public places; Indoor workplaces; Outdoor and quasi-outdoor environments	Implementation; Compliance; Enforcement	Ramachandran S, Bentley S, Casey E, Bentley JP. Prevalence of and factors associated with violations of a campus smoke-free policy: a cross-sectional survey of undergraduate students on a university campus in the USA. BMJ Open. 2020;10(3):e030504. doi:10.1136/bmjopen-2019-030504
**Ratschen et al., 2008**	United Kingdom: England	High-income	EURO	Indoor public places; Indoor workplaces	Development; Implementation; Enforcement; Compliance	Ratschen E, Britton J, McNeill A. Smoke-free hospitals – the English experience: results from a survey, interviews, and site visits. BMC Health Services Research. 2008;8:41. doi:10.1186/1472-6963-8-41
**Robertson et al., 2015**	New Zealand	High-income	WPRO	Indoor public places; Indoor workplaces; Outdoor and quasi-outdoor environments	Development; Implementation; Compliance; Enforcement	Robertson LA, Marsh L. Smoke-free policies in New Zealand public tertiary education institutions. Health Educ Res. 2015;30(2):347–58. doi:10.1093/her/cyv004
**Robertson et al., 2018**	Uganda	Low-income	AFRO	Indoor public places; Indoor workplaces; Outdoor and quasi-outdoor environments; Public transport	Development; Implementation; Compliance; Enforcement	Robertson L, Nyamurungi KN, Gravely S, Rusatira JC, Oginni A, Kabwama SN, et al. Implementation of 100% smoke-free law in Uganda: a qualitative study exploring civil society's perspective. BMC Public Health. 2018;18(1):927. doi:10.1186/s12889-018-5869-8
**Rosen et al., 2015**	Israel	High-income	EURO	Indoor public places; Indoor workplaces; Outdoor and quasi-outdoor environments; Public transport	Development; Implementation	Rosen B, Peled-Raz M. Tobacco policy in Israel: 1948–2014 and beyond. Israel Journal of Health Policy Research. 2015;4:12. doi:10.1186/s13584-015-0007-x

**Rozema et al., 2016**	Netherlands	High-income	EURO	Outdoor and quasi-outdoor environments	Implementation; Compliance; Enforcement	Rozema AD, Mathijssen JJP, Jansen MWJ, van Oers JAM. Schools as smoke-free zones? Barriers and facilitators to the adoption of outdoor school ground smoking bans at secondary schools. Tobacco Induced Diseases. 2016;14(1). doi:10.1186/s12971-016-0076-9
**Rozema et al., 2018**	Netherlands	High-income	EURO	Outdoor and quasi-outdoor environments	Implementation	Rozema AD, Mathijssen JJP, Jansen MWJ, van Oers JAM. Sustainability of outdoor school ground smoking bans at secondary schools: a mixed-method study. Eur J Public Health. 2018;28(1):43–9. doi:10.1093/eurpub/ckx099
**Satterlund et al., 2009**	United States	High-income	AMRO	Indoor public places; Indoor workplaces	Implementation; Compliance; Enforcement	Satterlund TD, Lee JP, Moore RS, Antin TM. Challenges to implementing and enforcing California's Smoke-Free Workplace Act in bars. Drugs Abingdon Engl. 2009;16(5):422–35. doi:10.1080/09687630802302872
**Satterlund et al., 2011**	United States	High-income	AMRO	Outdoor and quasi-outdoor environments	Development; Implementation; Enforcement	Satterlund TD, Cassady D, Treiber J, Lemp C. Strategies implemented by 20 local tobacco control agencies to promote smoke-free recreation areas, California, 2004-2007. Prev Chronic Dis. 2011;8(5):A111. PMID:21843414
**Satterlund et al., 2011**	United States	High-income	AMRO	Outdoor and quasi-outdoor environments	Implementation; Enforcement	Satterlund TD, Cassady D, Treiber J, Lemp C. Barriers to adopting and implementing local-level tobacco control policies. J Community Health. 2011;36(4):616–23. doi:10.1007/s10900-010-9350-6

**Satterlund et al., 2012**	United States	High-income	AMRO	Indoor public places; Indoor workplaces	Implementation; Enforcement	Satterlund TD, Lee JP, Moore RS. Changes in smoking-related norms in bars resulting from California's Smoke-Free Workplace Act. J Drug Educ. 2012;42(3):315–26. doi:10.2190/DE.42.3.d
**Sebrié et al., 2008**	Multiple	Lower-middle income; Upper-middle income; High-income	AMRO	Indoor public places; Indoor workplaces; Public transport	Development; Implementation	Sebrié EM, Schoj V, Glantz SA. Smoke free environments in Latin America: on the road to real change? Prev Control. 2008;3(1):21–35. doi:10.1016/j.precon.2007.09.001
**Shipley et al., 2008**	United Kingdom	High-income	EURO	Indoor public places; Indoor workplaces; Outdoor and quasi-outdoor environments	Implementation; Compliance; Enforcement	Shipley M, Allcock R. Achieving a smoke-free hospital: Reported enforcement of smoke-free regulations by NHS health care staff. J Public Health. 2008;30(1):2–7. doi:10.1093/pubmed/fdn004
**Sinha et al., 2011**	Multiple	Lower-middle income; Upper-middle income	SEARO	Indoor public places; Indoor workplaces; Public transport	Development; Implementation; Enforcement	Sinha DN, Narain JP, Kyaing NN, Rinchen S. WHO Framework Convention on Tobacco Control and its Implementation in South-East Asia Region. Indian Journal of Public Health. 2011;55(3):184-91. doi:10.4103/0019-557X.89949
**Skeer et al., 2004**	United States	High-income	AMRO	Indoor public places; Indoor workplaces	Compliance; Enforcement	Skeer M, Land ML, Cheng DM, Siegel MB. Smoking in Boston bars before and after a 100% smoke-free regulation: an assessment of early compliance. J Public Health Manag Pract JPHMP. 2004;10(6):501–7. doi:10.1097/00124784-200411000-00005

**Štěpánková et al., 2020**	Czech Republic	High-income	EURO	Indoor public places; Indoor workplaces	Development; Implementation	Štěpánková L, Kostelecká L, Stejskalová V, Kalvachová M, Králíková E. Hospital as a smoke-free workplace. Cent Eur J Public Health. 2020;28:S26–30. doi:10.21101/cejph.a6172
**Suarjana et al., 2020**	Indonesia	Upper-middle-income	SEARO	Indoor public places; Indoor workplaces; Outdoor and quasi-outdoor environments; Public transport	Development; Implementation; Compliance; Enforcement	Suarjana K, Astuti PAS, Putra IWGAE, Duana MK, Mulyawan KH, Chalidyanto D, et al. Implementation of smoke-free law in denpasar bali: Between compliance and social norms of smoking. J Public Health Res. 2020;9(3):246–54. doi:10.4081/jphr.2020.1747
**Tay et al., 2008**	New Zealand	High-income	WPRO	Outdoor and quasi-outdoor environments	Development	Tay S, Thomson G. What Wellington region city councillors think of smokefree outdoor places. N Z Med J. 2008;121(1276):15–28. PMID:18574506
**Tumwine et al., 2011**	Multiple	Low-income; Lower-middle income; Upper-middle income; High-income	AFRO	Indoor public places; Indoor workplaces; Outdoor and quasi-outdoor environments; Public transport	Development; Implementation	Tumwine J. Implementation of the Framework Convention on Tobacco Control in Africa: Current status of legislation. Int J Environ Res Public Health. 2011;8(11):4312–31. doi:10.3390/ijerph8114312
**Uang et al., 2016**	Multiple	Low-income; Lower-middle income; Upper-middle income; High-income	AFRO, AMRO, EMRO, EURO, SEARO, WPRO	Indoor public places; Indoor workplaces	Implementation; Compliance; Enforcement	Uang R, Hiilamo H, Glantz SA. Accelerated Adoption of Smoke-Free Laws After Ratification of the World Health Organization Framework Convention on Tobacco Control. Am J Public Health. 2016;106(1):166-171. doi:10.2105/AJPH.2015.302872

**Uang et al., 2017**	Colombia	Upper-middle income	AMRO	Indoor public places; Indoor workplaces; Outdoor and quasi-outdoor environments; Public transport	Development; Implementation; Enforcement	Uang R, Crosbie E, Glantz SA. Smokefree implementation in Colombia: Monitoring, outside funding, and business support. Salud Publica Mex. 2017;59(2):128–36. doi:10.21149/7884
**Ueda et al., 2011**	Japan	High-income	WPRO	Outdoor and quasi-outdoor environments	Development; Implementation; Enforcement	Ueda H, Armada F, Kashiwabara M, Yoshimi I. Street smoking bans in Japan: A hope for smoke-free cities? Health Policy. 2011;102(1):49–55. doi:10.1016/j.healthpol.2011.05.013
**Vardavas et al., 2011**	Greece	High-income	EURO	Indoor public places; Indoor workplaces; Public transport	Development; Implementation; Compliance; Enforcement	Vardavas CI, Dimitrakaki C, Schoretsaniti S, Patelarou E, Filippidis FT, Connolly GN, et al. The role of the non-smoker in enforcing smoke-free laws. J Public Health Policy. 2011;32(1):46–59. doi:10.1057/jphp.2010.45
**Vardavas et al., 2013**	Greece	High-income	EURO	Indoor public places; Indoor workplaces	Compliance; Enforcement	Vardavas CI, Agaku I, Patelarou E, Anagnostopoulos N, Nakou C, et al. Ashtrays and Signage as Determinants of a Smoke-Free Legislation's Success. PLoS ONE. 2013;8(9): e72945. doi:10.1371/journal.pone.0072945
**Verdonk-Kleinjan et al., 2013**	Netherlands	High-income	EURO	Indoor workplaces	Development; Compliance	Verdonk-Kleinjan WMI, Rijswijk PCP, de Vries H, Knibbe RA. Compliance with the workplace-smoking ban in the Netherlands. Health Policy. 2013;109(2):200–6. doi:10.1016/j.healthpol.2012.11.006

**Wahyuti et al., 2019**	Indonesia	Upper-middle income	SEARO	Indoor public places; Indoor workplaces; Outdoor and quasi-outdoor environments; Public transport	Development; Implementation; Compliance; Enforcement	Wahyuti W, Hasairin SK, Mamoribo SN, Ahsan A, Kusuma D. Monitoring compliance and examining challenges of a smoke-free policy in Jayapura, Indonesia. J Prev Med Pub Health. 2019;52(6):427–32. doi:10.3961/jpmph.19.240
**Wei et al., 2019**	China	Upper-middle income	WPRO	Indoor public places; Indoor workplaces; Public transport	Compliance; Enforcement	Wei Y, Borland R, Zheng P, Fu H, Wang F, He J, Feng Y. Evaluation of the Effectiveness of Comprehensive Smoke-Free Legislation in Indoor Public Places in Shanghai, China. Int J Environ Res Public Health. 2019;16(20):4019. doi:10.3390/ijerph16204019
**Widome et al., 2010**	United States	High-income	AMRO	Indoor public places; Indoor workplaces	Development; Implementation	Widome R, Samet JM, Hiatt RA, Luke DA, Orleans CT, Ponkshe P, et al. Science, prudence, and politics: The case of smoke-free indoor spaces. Ann Epidemiol. 2010; 20(6):428–35. doi:10.1016/j.annepidem.2010.03.004
**Wynne et al., 2018**	Multiple	Upper-middle income; High-income	AMRO, EURO, SEARO, WPRO	Indoor public places; Indoor workplaces; Public transport	Development; Implementation; Compliance; Enforcement	Wynne O, Guillaumier A, Twyman L, McCrabb S, Denham AMJ, Paul C, Baker AL, Bonevski B. Signs, Fines and Compliance Officers: A Systematic Review of Strategies for Enforcing Smoke-Free Policy. International Journal of Environmental Research and Public Health. 2018; 15(7):1386. doi:10.3390/ijerph15071386
**Xiao et al., 2013**	China	Upper-middle income	WPRO	Indoor public places; Indoor workplaces	Implementation	Xiao D, Wang C, Chen H, Hajek P. Making hospitals in China smoke-free: A prospective study of implementing the new standard. Nicotine Tob Res. 2013;15(12):2076–80. doi:10.1093/ntr/ntt098

**Yamada et al., 2015**	Japan	High-income	WPRO	Indoor public places; Indoor workplaces; Outdoor and quasi-outdoor environments	Development; Implementation; Compliance; Enforcement	Yamada K, Mori N, Kashiwabara M, Yasuda S, Horie R, Yamato H, et al. Industry Speed Bumps on Local Tobacco Control in Japan? The Case of Hyogo. J Epidemiol Jpn Epidemiol Assoc. 2015;25(7):496–504. doi:10.2188/jea.JE20150001
**Yunarman et al., 2021**	Indonesia	Upper-middle income	SEARO	Indoor public places; Indoor workplaces	Implementation; Compliance; Enforcement	Yunarman S, Munandar A, Ahsan A, Akbarjono A, Kusuma D. Opportunities and Challenges of Tobacco Control Policy at District Level in Indonesia: A Qualitative Analysis. Asian Pac J Cancer Prev APJCP. 2021;22(10):3055–60. doi:10.31557/APJCP.2021.22.10.3055
**Zasimova et al., 2019**	Russia	Upper-middle income	EURO	Indoor public places; Indoor workplaces; Outdoor and quasi-outdoor environments; Public transport	Compliance; Enforcement	Zasimova L. Analysis of non-compliance with smoke-free legislation in Russia. Int J Public Health. 2019;64(3):413–22. doi:10.1007/s00038-018-1198-z
**Zhang et al., 2019**	China	Upper-middle income	WPRO	Indoor public places; Indoor workplaces; Public transport	Implementation; Compliance; Enforcement	Zhang J, Cui X, Liu H, Han H, Cao R, Sebrie EM, et al. Public mobilisation in implementation of smoke-free Beijing: a social media complaint platform. Tob Control. 2019;28(6):705–11. doi:10.1136/tobaccocontrol-2018-054534
**Zhou et al., 2016**	Multiple	Upper-middle income; High-income	AMRO, EURO, WPRO	Indoor public places; Indoor workplaces	Development; Implementation; Compliance; Enforcement	Zhou L, Niu L, Jiang H, Jiang C, Xiao S. Facilitators and Barriers of Smokers' Compliance with Smoking Bans in Public Places: A Systematic Review of Quantitative and Qualitative Literature. Int J Environ Res Public Health. 2016;13(12):1228. doi:10.3390/ijerph13121228

© **2024 Teed J.A. et al.**
